# Retinal Vascular Signs as Screening and Prognostic Factors for Chronic Kidney Disease: A Systematic Review and Meta-Analysis of Current Evidence

**DOI:** 10.3390/jpm11070665

**Published:** 2021-07-15

**Authors:** Michael Aronov, Raviv Allon, Danielle Stave, Michael Belkin, Eyal Margalit, Ido Didi Fabian, Barak Rosenzweig

**Affiliations:** 1 SPRING Biomed Vision Ltd. 8, Haneviim St., Haifa 3350109, Israel; ravivallon@gmail.com (R.A.); daniellestave@gmail.com (D.S.); eyal@springvisionbiomed.com (E.M.); 2 The Goldschleger Eye Institute, Sheba Medical Center, Tel Hashomer 5262000, Israel; belkin@netvision.net.il (M.B.); didifabian@gmail.com (I.D.F.); 3 Sackler Faculty of Medicine, Tel Aviv University, Tel Aviv 69978, Israel; 4 Department of Urology, Sheba Medical Center, Tel Hashomer 5262000, Israel; barak22@gmail.com

**Keywords:** chronic kidney disease, end-stage renal disease, fundus photography, glomerular filtration rate, retinal vascular diameter, diabetic retinopathy

## Abstract

Background: The substantial burden of kidney disease fosters interest in new ways of screening for early disease diagnosis, especially by non-invasive imaging. Increasing evidence for an association between retinal microvascular signs and kidney disease prompted us to investigate the relevant current literature on such an association systematically by performing a meta-analysis of our findings. Methods: We scrutinized the current literature by searching PubMed and Embase databases from for clinical studies of the association between retinal microvascular signs and prevalent or incident kidney disease. After excluding cases that did not meet our criteria, we extracted relevant data from 42 published studies (9 prospective, 32 cross-sectional, and 1 retrospective). Results: Our investigation yielded significant associations between retinal vascular changes (including retinopathy and retinal vascular diameter) and kidney dysfunction (including chronic kidney disease (CKD), end-stage renal disease (ESRD), albuminuria, and estimated glomerular filtration rate (eGFR) decline). According to our meta-analysis, retinopathy was associated with ESRD (hazard ratio (HR) 2.12 (95% confidence interval CI; 1.39–3.22)) and with CKD prevalence in the general population (odds ratio (OR) 1.31 (95% CI; 1.14–1.50)), and specifically in type 2 diabetic patients (OR 1.68 (95% CI; 1.68–2.16)). CRAE was associated with prevalent CKD (OR 1.41 (95% CI; 1.09–1.82)). Conclusions: Our findings suggest that the retinal microvasculature can provide essential data about concurrent kidney disease status and predict future risk for kidney disease development and progression.

## 1. Introduction

Chronic kidney disease (CKD) is a major public health problem that often results in adverse renal and cardiovascular outcomes and premature death. In 2016, the global prevalence of CKD across all age groups was estimated to be 13.4% [1]. There is a strong link between kidney disease and vascular risk factors, such as diabetes and hypertension, which are known to have major microvascular components [2,3,4]. The known concurrence between diabetic retinopathy (DR) and diabetic nephropathy (DN) suggests that the same mechanisms might be the underlying cause of the development of both ocular and renal complications of diabetes [5]. 

CKD has long been medically challenging owing to the late appearance of its clinical symptoms and the late initiation of standard therapies. Visualization of the retinal microvasculature provides a unique opportunity to study the systemic microvasculature noninvasively [6]. Identifying new predictors of a microvascular disease can be helpful for early individual risk stratification, which may in turn facilitate a better opportunity for the timely application of effective interventions [5].

The most frequently observed retinal microvascular parameters are retinal vascular diameter and retinopathy. The latter is a disease caused by longstanding ischemia and characterized by typical findings on retinal images, including microaneurysms, hemorrhages, hard and soft exudates, and other vascular abnormalities [7], and multiple studies have examined its relationship to kidney and other systemic diseases [6,8,9]. However, evidence derived from other retinal vascular signs, both qualitative and quantitative, and including vessel tortuosity, branching angles of vessels, fractal dimensions (Df), and arterio-venous (AV) nicking, has been accumulating along with the development of different imaging modalities, both existing (fundus photography and fluorescein angiography (FA)) and new, such as optical coherence tomography (OCT) and OCT angiography (OCTA).

In this study, we systematically searched the literature for associations between the retinal microvasculature and both the incidence and the prevalence of kidney disease. Our aim was to investigate the retinal microvasculature as a potential screening and diagnostic source for early detection of kidney disease. 

## 2. Materials and Methods

### 2.1. Search Strategy and Study Selection

In conducting this study, we adhered to the guidelines of the Meta-Analysis of Observational Studies in Epidemiology [10]. Literature searches were carried out in PubMed and Embase using the combination of search terms provided in Appendix A, which also contains a detailed protocol of our study. Our study population consisted of patients without CKD who developed incident CKD, patients with CKD who developed incident end-stage renal disease (ESRD), and patients with concurrent CKD or ESRD, and early kidney dysfunction, with signs including albuminuria, proteinuria, elevated serum creatinine, and decline in eGFR. For ophthalmic imaging, we included fundus color photography, OCT, and OCTA as the intervening modalities. For the included studies, we reported odds ratios (ORs), HRs, and RRs, all with 95% confidence intervals (CI) or regression coefficients (β) with the standard error (SE).

Excluded from our research were case reports, studies published only as abstracts or presented in conferences without full subsequent publication, studies with fewer than 10 patients, and review papers.

### 2.2. Data Extraction 

Each article was examined by two independent reviewers. In the case of a disagreement, the article was discussed by the reviewers until a consensus was achieved. The following data were extracted: author, year of publication, study type (cohort or cross-sectional, prospective or retrospective), number of participants, mean or median age of participants, gender (% male), risk factors used in regression model, follow-up period, ORs/HRs/β with CI or SE for the relationship between various retinal microvascular signs and CKD diagnosis, and related characteristics, including eGFR and albuminuria. For most of the articles we used the clinical practice guidelines of The Kidney Disease: Improving Global Outcomes (KDIGO) organization (2012) [11].

### 2.3. Retinal Microvascular Signs

We observed a wide range of retinal vascular signs by using different devices, as defined briefly in the following:

1. Fundus photography-derived signs:
  - Central retinal arteriolar equivalent (CRAE) and central retinal venular equivalent (CRVE), measured as the average of the diameter of the largest 6 arterioles and largest 6 venules, respectively [9].
  - Arterio-venous ratio (AVR), i.e., the ratio of CRAE to CRVE [12].
  - Simple tortuosity, derived from the ratio of actual path length to straight-line length of a retinal vessel segment; or curvature tortuosity, derived from the integral of the curvature squared along the path of the vessel, and normalized by the total path length.
  - Focal narrowing over a constricted area of ≤⅔ of the widths of proximal and distal vessel segments [2].
  - Fractal dimension (Df), used to quantify the branching architecture of the retinal vasculature using the box-counting method [6].
  - Optimality ratio, a measure of blood-flow loss in bifurcations related to endothelial dysfunction; optimality deviation measures the extent to which the optimality ratio deviates from the theoretically predicted optimum.
  - Branching angle, the first angle between two daughter vessels at each vascular bifurcation [6].
  - Retinopathy, considered to be present if any characteristic lesion (microaneurysms, hemorrhages, cotton-wool spots, intraretinal microvascular abnormalities, hard exudates, venous beading or new vessels) was present [6]. A retinopathy severity score was assigned according to the modified Airlie House classification system [8] or the Early Treatment Diabetic Retinopathy Study (ETDRS) score [9].
2. OCTA-derived signs:
  - Capillary flow density or capillary perfusion density of the retinal superficial capillary plexus, the retinal deep capillary plexus, and the choriocapillary layer [5].
  - Capillary vessel density, i.e., total length of the perfused vasculature per unit area in the region of measurement [13].

### 2.4. Evaluation of Renal Disease

1. eGFR was calculated from serum creatinine using the Modification of Diet in Renal Disease (MDRD) equation [14] or the Chronic Kidney Disease Epidemiology Collaboration (CKD-EPI) equation [6].
2. Albuminuria was calculated from spot urine specimens and was defined as:
  - Albumin to creatinine ratio (ACR) > 17 mg/g in men and > 25 mg/g in women [15,16,17].
  - ACR = 30–300 µg/mg was considered as microalbuminuria, and ACR > 300 µg/mg was considered as macroalbuminuria [18,19,20].
  - ACR > 2.5 mg/mmol for men and ACR > 3.5 mg/mmol for women were considered as albuminuria [21].
3. Incident CKD
  - Defined by Kidney Disease Outcomes Quality Initiatives (KDOQI) criteria [22], or by the KDIGO criteria [11].
  - Or, defined as eGFR < 60 mL/min/1.73 m^2^ accompanied by a decrease in eGFR of at least 25% over the follow-up period among subjects free of CKD at baseline, or as eGFR < 45 mL/min/1.73 m^2^ accompanied by a decrease in eGFR of at least 25% over the follow-up period among subjects free of CKD at baseline, or as a composite of incident CKD and/or rapid decline in eGFR (annual eGFR rate reduction of > 3 mL/min/1.73 m^2^/year) [6].
4. Incident ESRD was defined as the initiation of chronic dialysis therapy or kidney transplantation [12].
5. Prevalent ESRD was defined as eGFR of < 15 mL/min, or a serum creatinine level ≤ 5.7 mg/dL, or a new requirement of kidney failure treatment [8].

### 2.5. Statistical Analysis

The meta-analyses were performed with R-studio software, version 1.3.959 (2020). The primary goal of this meta-analysis is to compute the aggregative effect size between retinal microvascular abnormalities and kidney-related diseases. The following data were gathered from the publications that met inclusion and exclusion criteria: sample size, sex and age of the cohort, the exact definition of kidney parameters, adjustment parameters, and the effect size reported. Several meta-analyses were conducted, according to the definitions of retinal microvascular abnormalities and kidney-related diseases. Two main types of effects were obtained: (1) Hazard Ratio—which indicates the probability of accumulated survival over time. (2) Odds Ratio—which indicates the probability of kidney-related diseases’ occurrence. In each meta-analysis, we computed the aggregative effect size (OR or HR), its *p*-value, and also the 95% confidence interval with the lower limit [LL] and upper limit [UL]. Heterogeneity between the studies in each meta-analysis was assessed using the *Q* statistic. *Q* is a chi-square statistic (with degrees of freedom equal to *k* – 1), which reflects variability among effect estimates due to true heterogeneity, rather than sampling error. To visualize the results, we present forest plots for each meta-analysis. 

### 2.6. Risk of Bias

Risk of bias was assessed by means of the Quality In Prognosis (QUIPS) tool [23]. QUIPS is a standard tool used by Cochrane to review cohort studies evaluating predictive factors in prognosis or diagnosis. Risk of bias for each study is assessed in six domains: study participation, study attrition, prognostic factor measurement, outcome measurement, study confounding, and statistical analysis and report. The overall risk of bias score for each study was assessed according to that proposed by Lazzerini et al [24]. A low risk of bias was given if all six domains were scored as low, or if not more than two moderate or unknown risks of bias were identified. Moderate risk of bias was given when three or fewer risk of bias domains were scored moderate, or unknown, in combination with no high risk of bias. Moderate was also given when one domain was scored as a high risk of bias in combination with one at most with moderate risk of bias or unknown risk. A high risk of bias was given when two or more domains scored a high risk of bias, or four or more with moderate or unknown risks of bias. Risk of bias was assessed by two independent reviewers; in the case of disagreement, the article was discussed until consensus was reached. 

## 3. Results

A literature search yielded 1331 articles from PubMed and 535 from Embase. Following exclusion of duplicates, 1834 records were identified. A review of the records yielded 118 full-text articles for the assessment of eligibility, of which only 23 met our inclusion criteria. 

Following cross-referencing, 42 studies (9 prospective cohorts, 32 cross-sectional studies, and 1 retrospective study) met our eligibility criteria, and all 42 were included in this research. Of the 42 studies, 6 were included in the meta-analysis, in 4 different evaluations. 

**Figure A1:**
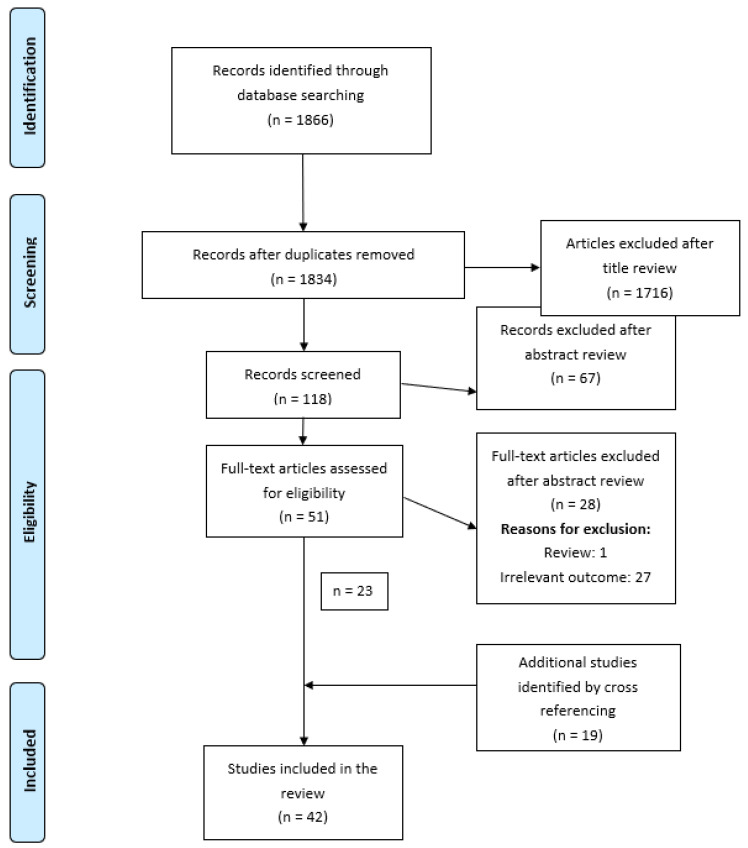
PRISMA.

The Preferred Reporting Items for Systematic Reviews and Meta-Analyses (PRISMA) flow diagram (Figure A1 in Appendix A) depicts the study selection.

### 3.1. Risk of Bias

The results of the scoring of the methodological quality of the studies are shown in Supplementary Table S1. Overall, the methodological quality was good: 22 studies were scored as having low risk of bias (52.38%); 11 studies were scored as having moderate risk of bias (26.19%); and 9 studies were scored as having high risk of bias (21.42%).

### 3.2. Retinal Microvascular Signs and CKD

#### 3.2.1. Retinal Microvascular Signs and Incident CKD

Three prospective studies, which included high-resolution retinal photographs, followed patients with normal kidney functions for incident CKD (Table 1). Yip et al. reported that narrower CRAE, wider CRVE, and retinopathy were associated with incident CKD [6]. Yau et al. reported that narrower CRAE was associated with incident CKD in whites [9]. Finally, Sabanayagam et al. reported that retinal vascular diameter was not associated with incident CKD [25].

#### 3.2.2. Retinal Microvascular Signs and Deterioration of CKD

Three prospective studies followed patients with CKD for associations between retinal vascular parameters and ESRD (Table 2). Two of those studies reported a statistically significant association between the presence of retinopathy and incident ESRD in CKD patients (Table 2) [8,12]. One of them reported that when adjusted for age, systolic blood pressure, race, diabetes, body mass index (BMI), smoking status, clinical site, and estimated glomerular filtration rate (eGFR), retinopathy was associated with incident ESRD. When additionally adjusted for 24 h urine protein, the association was not statistically significant. In addition, patients with ungradable images showed the strongest associations with incident ESRD across all models, including the fully adjusted model [12]. 

Two studies reported that measures of retinal vascular diameter were associated with incident ESRD [12,26].

#### 3.2.3. Retinal Microvascular Signs and Concurrent CKD

**Figure 1:**
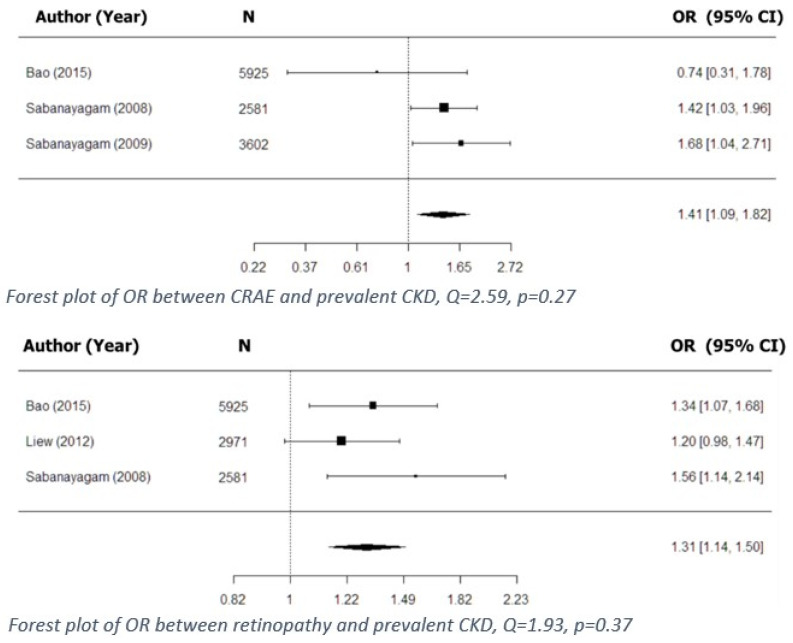
Cross-sectional associations between retinal vascular signs and kidney dysfunction.

A total of 11 studies examined cross-sectional associations between retinal vascular parameters and prevalent CKD (Table 3). The above-mentioned associations based on retinal vascular diameter were examined in 8 studies, 3 of which were included in our meta-analysis (Figure 1), which showed that narrower CRAE was associated with prevalent CKD.

Sabanayagam et al. reported that narrower CRAE was associated with prevalent CKD in the Singapore Prospective Cohort Program, which was later replicated in another independent cohort, the Singapore Eye Malay Study (data not shown). In both cohorts, the association was found to be even stronger when narrower CRAE was combined with the presence of hypertension [14]. Sabanayagam et al. also reported, in two different studies, that CRAE per standard deviation (SD) decrease was associated with prevalent CKD [14,15]. Liew et al. reported that a wider CRVE (quintile 5 vs. quintiles 1–4) was associated with prevalent CKD, and that when further stratified by the presence of diabetes, the association remained significant only in diabetic patients [27]. Two studies reported on the retinal vascular diameter as a discriminating factor between mild and moderate/severe forms of CKD: Gu et al. reported that narrower CRAE (<150 µm) discriminated CKD stage 1 from CKD stage 2 and beyond [29], while Ooi et al. reported that both narrower CRAE and narrower CRVE distinguished CKD stages 1 to 2 from stages 3–5. The ORs reported in these two studies were 3.48 (95% CI; 1.67–7.24) and 4.0 (95% CI; 1.86–8.58), respectively [30].

Of four studies that examined cross-sectional associations for prevalent CKD based on retinopathy or signs of retinopathy (Table 3), three were included in the meta-analysis (Figure 1) [3,15,27]. All three showed that retinopathy is associated with prevalent CKD. In addition, Wong et al. showed that the presence of retinopathy, or specific signs of retinopathy, was associated with the development of renal dysfunction [2].

Five studies examined cross-sectional associations between retinal microvascular signs and prevalent CKD that were based on parameters other than retinal vascular caliber or retinopathy (Table 3). Wong et al. reported that arteriovenous (AV) nicking was associated with prevalent CKD [2]. Lim et al. reported that AV nicking showed a nonsignificant trend towards an association with prevalent CKD [16]. Sng et al. reported that both lower (quintile 1 vs. 4) and higher (quintile 5 vs. 4) Df were associated with prevalent CKD [31]. The study of Vadala et al. was the only one that used OCT/OCTA as its preferred imaging modality. Decreased retinal thickness, decreased choroidal thickness, and superficial and deep choroidal vascular densities were all found to be associated with prevalent CKD [13]. 

#### 3.2.4. Retinal Microvascular Signs and Concurrent ESRD

Two studies examined cross-sectional associations between retinal microvascular signs and prevalent ESRD (Table 4). Yip et al. reported that retinopathy was found to be associated with prevalent ESRD [8]. Grunwald et al. reported on associations between changes in retinal vascular diameter and worsening of retinopathy, and prevalent ESRD or a 50% decline in eGFR between initial and follow-up photography. Worsening of retinopathy between initial and follow-up photography was associated with prevalent ESRD only in the univariate analysis [32].

### 3.3. Retinal Microvascular Signs and Concurrent eGFR

Six studies examined cross-sectional associations between retinal microvascular signs and change in eGFR (Table 5). Of these, three studies reported significant relationships between eGFR and retinal vascular diameter, and all three reported that CRAE, but not CRVE, was associated with eGFR decline [16,29,33].

Three studies reported on relationships between eGFR and the presence of retinopathy or specific signs of retinopathy (including retinal hemorrhages, microaneurysms, and hard or soft exudates). Two of these studies reported on positive associations between retinopathy or signs of retinopathy and changes in eGFR [34] or in the eGFR slope [35], but the third study did not [32].

Associations between eGFR and retinal microvascular signs other than retinal vascular diameter or retinopathy were reported in three studies (Table 5) [16,26,34]. One of them, a study by Lim et al., reported that Df and arteriovenous nicking(AVN) were associated with eGFR decline [16].

### 3.4. Retinal Microvascular Signs and Concurrent Albuminuria

A total of five studies reported on cross-sectional associations between retinal microvascular signs and albuminuria (Table 6). All of the included studies reported on associations between retinal vascular diameter and albuminuria. Two reported on an association with narrower CRAE (when examined as a categorical variable) [3,15,20]. Awua-larbi et al. demonstrated a U-shaped pattern of association between CRAE and albuminuria, an association that was shown by CRAE in quintiles 1 and 5 (compared to quintile 3) [20]. Garcia-Ortiz et al. and Sabanayagam et al. reported that both wider CRVE and narrower CRAE were associated with albuminuria. Several studies reported an association between AVR and albuminuria [3,15,36].

Both Bao et al. and Sabanayagam et al. reported that retinopathy was associated with the presence of albuminuria (Table 6) [3,15], while Lim et al. reported that Df, focal arteriolar narrowing (FAN), and AVN were all associated with albuminuria (Table 6) [16].

### 3.5. Retinal Microvascular Signs and Renal Dysfunction in Diabetes

#### 3.5.1. Retinal Microvascular Signs and Incident CKD

One study reported on prospective associations between retinal vascular signs and incident CKD in diabetic patients. This was a population-based cohort study that followed 557 patients, 51% to 51.8% males, with “younger onset” type 1 diabetes, who were diagnosed and had received insulin therapy before the age of 30. Follow-up lasted for 20 years. In multivariable analysis, wider CRVE—both in continuous and in categorical analysis—was found to be associated with incident CKD (risk ratio (RR) 1.51 (95% CI; 1.05–2.17), RR 1.22 (95% CI; 1.04–1.44), respectively). When wider CRVE was combined with the presence or absence of retinopathy, the associations became stronger. RRs for the presence of any retinopathy or proliferative retinopathy were 1.7 (95% CI; 1.19–2.43) and 1.77 (95% CI; 1.09–2.9), respectively. Interestingly, when wider CRVE was combined with the absence of retinopathy, the association became even stronger (RR 3.05 (95% CI; 1.05–8.92)) [37].

#### 3.5.2. Retinal Microvascular Signs and Deterioration of CKD 

Two studies reported on prospective associations between retinal microvascular signs and incident ESRD (Table 7). Both studies used high-resolution digital cameras, and one of them used FA. 

Yip et al. examined the relationship between retinal microvascular signs, including CRAE, CRVE, Df, and retinopathy, and the incidence and prevalence of ESRD in a multi-ethnic Asian population comprised of both diabetic and nondiabetic patients. The study utilized data from two population-based cohorts (SiMES and SP2 datasets). Retinopathy, but not other signs, was associated with ESRD when stratified by the status of diabetes [8]. Lee et al. followed 51 patients with diabetic retinopathy in a population-based cohort for 2 years, assessing their retinal metabolic status via FA. A nonperfusion area of more than 10 disc areas on FA was strongly associated with incident ESRD. Neovascularization and vitreous hemorrhage were associated with incident ESRD only in univariate analysis [38].

#### 3.5.3. Retinal Microvascular Signs and Concurrent CKD

**Figure 2:**
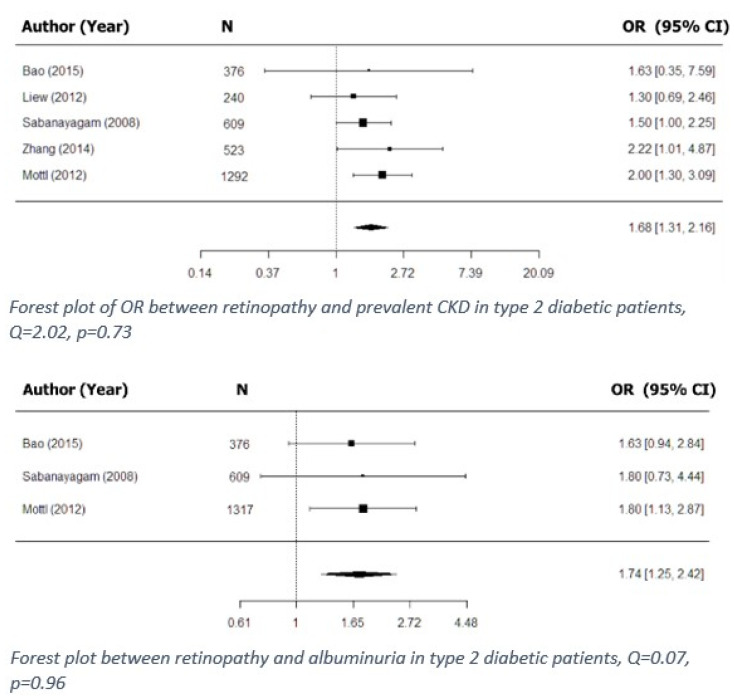
Cross-sectional associations between retinopathy and kidney dysfunction in type 2 diabetes.

Cross-sectional associations between retinal microvascular signs and prevalent CKD in diabetic patients were reported in seven studies (Table 8). Of these, six examined such associations based on the presence of retinopathy, and five of the six were included in the meta-analysis (Figure 2) [3,15,17,19,27]. The findings showed that retinopathy was associated with prevalent CKD in type 2 diabetes.

Mottl et al. reported that an association between retinopathy and prevalent CKD in type 2 diabetic patients was found only in non-Hispanic blacks, in obese patients, and in patients not using renin-angiotensin-aldosterone (RAAS) blockers [19]. Zhang et al. and Wong et al. reported that in type 2 diabetic patients, both proliferative and nonproliferative DR were associated with prevalent CKD. When the presence of retinopathy was combined with hypertension, the association became stronger. Interestingly, in patients with type 2 diabetes, proliferative DR without hypertension was most strongly associated with prevalent CKD [17,37].

Parameters other than retinopathy and their associations with prevalent CKD in diabetic patients were examined in three out of the seven included studies. The only significant associations were described by Bao et al., who reported that when retinal vascular signs and prevalent CKD or albuminuria were examined, associations of CRAE and CRVE with kidney function were significant [3].

#### 3.5.4. Retinal Microvascular Signs and Diabetic Nephropathy

Three studies reported on cross-sectional associations between retinal microvascular signs and DN in type 1 diabetic patients (Table 9). All three were conducted on the Danish Cohort of Pediatric Diabetes (DCPD). Note that in all three studies, DN has been defined as macroalbuminuria, renal transplantation, or renal replacement therapy. Studies defining DN as albuminuria only are included in another part of this article.

Broe et al. reported on statistically significant associations between DN and both CRAE and CRVE [18]. In another study, Broe et al. reported an association between Df and DN [40]. Rasmussen et al. reported that the arteriolar branching coefficient, a measure of the angle between two daughter branches, was associated with DN [41].

#### 3.5.5. Retinal Microvascular Signs and Concurrent eGFR in Diabetes

Three studies examined cross-sectional associations between retinal vascular parameters and concurrent eGFR decline in diabetic patients (Table 10). In one of them, Edwards et al. described a single positive association between soft exudates, hard exudates, and microaneurysms and eGFR decline during the follow-up period [34].

#### 3.5.6. Retinal Microvascular Signs and Albuminuria in Diabetes

A total of 13 studies examined cross-sectional associations between retinal vascular signs and concurrent albuminuria in diabetic patients (Table 11). Of these, six reported on associations between retinal vascular diameter and albuminuria. Benitez et al. reported that when blood-vessel diameters were measured in an extended zone (with smaller vessels included in the measurement), the mean retinal arteriolar diameter was found to be associated with a high risk for albuminuria. A larger mean venular width showed a similar trend towards a high risk for albuminuria [42]. In another study, Benitez et al. reported that both the venular tortuosity and the ratio of venular length to diameter (LDR) were associated (including prospective associations) with increased albuminuria [43]. Grauslund et al. reported that narrower CRAE was associated with albuminuria (as a continuous variable). However, when examined as a categorical variable, the association was significant only in the age- and sex-adjusted model [44]. Awua-larbi et al., Keel et al., and Bao et al. reported that narrower CRAE was associated with albuminuria in type 2 diabetic patients [3,20,21].

Out of 13 included studies, associations between retinopathy and albuminuria in diabetic patients were examined in four, of which three were cross-sectional and were included in the meta-analysis (Figure 2) [3,15,19], and one was retrospective. The meta-analysis showed that retinopathy was associated with the presence of albuminuria in type 2 diabetic patients. 

Mottl et al. reported that the presence of any retinopathy and of moderate to severe retinopathy was associated with albuminuria in type 2 diabetic patients [19]. Ha M. et al. performed a retrospective study on type 2 diabetic patients with DR, which required pan-retinal photocoagulation, in order to investigate potential risk factors for diabetic nephropathy. Vitreous hemorrhage was the only parameter associated with albuminuria [45].

Three studies reported on cross-sectional associations between albuminuria in diabetic patients based on AVR. Whereas Bao et al. and Keel et al. did not find a significant association between AVR and albuminuria in diabetic patients [3,21], Grauslund et al. reported that AVR was associated with albuminuria, both as a continuous and as a categorical variable, in type 1 diabetic patients [44].

Retinal vascular parameters other than retinal vascular diameter and retinopathy were reported in two articles. Grauslund et al. reported a trend towards an association between Df and albuminuria in type 1 diabetic patients [46]. Sasongko et al. reported that the arteriolar tortuosity index was associated with increased albuminuria in patients with type 1 diabetes [47].

Out of 13 studies included in this section, two reported cross-sectional associations for albuminuria based on findings seen on OCT/OCTA. Cankuratan et al. reported that vessel densities in superficial and deep capillary plexuses in the fovea and in parafoveal and perifoveal areas were associated with albuminuria [5]. Garrido et al. reported that central macular as well as inner nasal, inner superior, inner temporal, and inner inferior thicknesses were associated with albuminuria in diabetic patients [48].

## 4. Discussion

The most common causes of CKD are diabetes and hypertension [49], both of which have early manifestations in microvascular damage [50,51,52,53]. In a similar manner, retinopathy, a major complication of diabetes, is a sign of damaged retinal microvasculature caused by longstanding ischemia [54]. Unsurprisingly, therefore, in this review, we found strong evidence for associations between retinopathy (and specifically DR) and CKD in diabetic patients. We found both prospective and cross-sectional associations between kidney dysfunction (including CKD and ESRD) and retinopathy in both type 1 and type 2 diabetic patients [2,8,17,19,37]. Those findings were further supported by our meta-analysis, which showed that retinopathy is associated with prevalent CKD in type 2 diabetic patients (Figure 2). Furthermore, retinopathy, as well as individual signs of retinopathy (hard exudates and microaneurysms), was found to be associated with signs of early renal dysfunction, including eGFR decline [34] and the presence of albuminuria, in diabetic patients (Figure 2). In some studies, analysis of the total population was further stratified by taking confounding factors (such as hypertension) into account. Thus, for example, Zhang et al. reported that the association found between non-proliferative DR and prevalent CKD in type 2 diabetic patients, when stratified by status of hypertension, became even stronger [17]. It follows, therefore, that retinal screening in diabetic patients could have a double purpose, i.e., not only vision loss prevention, but also potential prediction of kidney disease development and progression, especially in hypertensive patients. 

Hypertension and diabetes were not the only factors associated with CKD. Mottl et al., reporting on the relationship between retinopathy and prevalent CKD in type 2 diabetic patients, found that these associations were significant in obese patients, in non-Hispanic black patients, and in patients not using RAAS blockers, but not in the overall study population without such potential confounders [19]. While, for most of the studies included in our review, the findings had been adjusted for common risk factors, in most of them, the study populations had not been stratified for potential confounders such as those mentioned, or for others, including gender, family history, age, smoking status, and more. 

Retinopathy was found to be associated with kidney disease not only in diabetic patients, but also in the general population. Our review exposed multiple cross-sectional and prospective associations between the presence of retinopathy and kidney disease. Furthermore, not only our meta-analysis but also individual studies showed that retinopathy is associated with prevalent CKD (Figure 1) in the overall population [6]. It seems, therefore, that diabetes simply represents a separate case of generalized microvascular dysfunction. In some studies, however, when further stratified by diabetes status, the association between CKD and retinopathy remained significant only in diabetic patients [12].

Interestingly, in some studies, the severity of retinopathy was shown to be associated with the severity of kidney function only during the follow-up period of that study [32]. In addition, patients whose retinal photographs were ungradable manifested the strongest associations with the incidence of ESRD [12]. Ocular media opacities, including cataracts, corneal opacities, and vitreous hemorrhages, as well as retinal detachment and poor pupillary dilatation, were all found capable of causing poor quality of retinal images, suggesting that the pathologies in those eyes are more severe than in eyes with gradable retinal images [12]. Therefore, ungradable fundus images should not be disregarded, as they may provide valuable information on the status of patients’ systemic diseases in general, and specifically of kidney-related diseases.

Even more than retinopathy, the most commonly discussed parameter in this review (and generally in the literature) is the retinal vascular diameter, which includes CRAE, CRVE, and AVR. Retinal arteriolar narrowing has been hypothesized to represent dysregulation of the RAAS system, as well as overexpression of endothelin, a potent vasoconstrictor that was postulated to be associated with sclerotic renal changes and kidney disease progression [55,56,57]. Furthermore, kidney biopsies of individuals with type 1 diabetes have shown that a narrower retinal arteriolar caliber is morphologically related to extracellular matrix accumulation, leading ultimately to a decrease in eGFR and CKD [58,59]. Larger venular diameters and increased blood flow are reportedly associated with diabetes status [60,61,62,63,64,65]. In addition, retinal venular widening and DR have been postulated to occur as a result of endothelial damage and inflammatory processes [66]. Clinically, retinal venular widening and DR represent thickening of the basement membrane, as well as increased leakage, which is also observed in CKD [67]. Altogether, the described processes point to a link between retinal arteriolar narrowing, retinal venular widening, decreased AVR, and kidney dysfunction. 

Yau et al. reported that narrower CRAE was associated with incident CKD, albeit only in whites [9], and Yip et al. reported an association between wider CRVE and incident CKD [6]. For the case of incident ESRD, there was only one study in which narrower CRAE was reported to be associated with incident ESRD and another one reporting that increased AVR was associated with ESRD, while other studies did not find similar associations [8,12,26]. While prospective studies have difficulties in finding associations between retinal vascular caliber and CKD, such associations are more readily found in cross-sectional studies. Our meta-analysis showed that CRAE is indeed associated with prevalent CKD (Figure 1). It therefore seems that retinal vascular caliber could be used as a prognostic factor for disease progression and severity. This possibility is supported by both Gu et al. and Ooi et al., who found that retinal vascular diameter can discriminate between early and late CKD [29,30]. One study reported an association between wider CRVE and incident CKD in type 1 diabetic patients on a long-term follow-up (20 years) [37]. Such a finding provides a clue as to the time needed for follow-up before statistically significant associations can be seen. 

Signs of kidney dysfunction, such as the presence of albuminuria or eGFR decline and their association with retinal vascular diameter, were examined and discussed in several reports. Individual studies showed statistically significant associations between retinal vascular diameter and albuminuria [29,33].

The literature does not offer a possible explanation for the discrepancy between the ability of prospective studies to predict CKD based on retinal vascular diameter and the abundance of cross-sectional studies showing associations between retinal vascular diameter and early kidney dysfunction (including albuminuria and eGFR decline). A similar association was mentioned in a recently published systematic review that examined associations between the retinal microvasculature and cardiac-related diseases. In that study, Allon et al. reported that while retinal vascular diameter failed to predict heart failure-related events, an abundance of cross-sectional associations between retinal vascular diameter and structural heart changes was observed in asymptomatic patients. Those changes included left ventricular hypertrophy, lower left ventricular systolic and diastolic functioning, and lower ejection fraction, leading those authors to suggest that asymptomatic patients who are at risk should be screened, and in some cases even treated with commonly accepted regimens in order to prevent deterioration to clinically significant heart failure [68]. Perhaps, according to similar reasoning, asymptomatic patients who are at risk for developing CKD should be screened for changes in their retinal vascular diameter and, depending on the findings, potentially treated to slow down progression of their condition. 

One study reported on an association between retinal vascular diameter and high risk for the development of albuminuria in type 1 diabetic adolescents. Interestingly, this association became significant only when smaller retinal arteriolar diameters were measured [42]. This highlights the importance of studying smaller retinal vessels and their possible associations with systemic diseases.

Retinal microvascular signs other than retinopathy and retinal vascular diameter have been examined in multiple studies, with mixed results. Perhaps the most controversial sign is fractal dimension (Df), a measure of the branching architecture of the retinal vascular tree. Significant positive associations between Df and CKD, eGFR decline, albuminuria, and DN were found in some studies [8,16,39,46], while no such associations were found in others [16,31,40]. Currently, the added value of such signs over retinal vascular diameter or retinopathy is unclear; however, as suggested by Allon et al., retinal vascular health is maintained not only by adequate retinal vascular diameter, but also by optimal vascular branching architecture as reflected by signs such as Df, AVN, and tortuosity [68]. One way to view the retinal microvasculature is to consider that it provides clues about the architectural health of the retinal vascular network. Each such sign attests to a disruption of the overall vasculature architecture, reflecting a pathological process caused, for example, by ischemia, and if such findings are disregarded, more advanced damage can occur, ultimately leading to retinopathy, CKD, or other ischemic conditions [69,70,71]. Furthermore, the exact contribution of each such sign to the disruption of healthy vascular architecture is hard to measure and to interpret. Future studies should be designed in a way that measures the additive effect of microvascular signs such as Df, AVN, and tortuosity. Such studies should adjust their findings for retinal vascular diameter and retinopathy in order to pinpoint their individual contribution and prevent potentially biased results. Furthermore, in the era of artificial intelligence, where algorithms based on machine learning and deep learning are becoming increasingly more popular for analysis of the retinal vasculature, the architecture of retinal branching should be studied more intensively for associations with kidney diseases, as well as other systemic diseases. As mentioned by both Spaide et al. and Gerendas et al., the quantity of potential retinal-disease biomarkers that suggest different disease origins and types is overwhelming [72,73]. That being the case, we should analyze possible associations with more advanced artificial intelligence-based tools, which could shed light on previously unknown associations.

Besides studies that used high-resolution digital traditional fundus cameras, four of the studies included in this review employed other types of imaging, three of them using OCTA [5,13,48] and one using FA [38]. Lee et al., using FA, found that nonperfusion of more than 10 disc areas in the retina was strongly associated with albuminuria in diabetic patients [38]. FA is an invasive diagnostic tool that detects real-time changes in retinal perfusion status. A new noninvasive perfusion detector could replace FA and revolutionize the field of retinal imaging. 

Our study has certain limitations. First, the reports used for our meta-analysis employed different methods of statistical analysis. While some did not use multivariate regression analysis, others adjusted for various factors such as diabetes, hypertension, age, gender, obesity, and ethnicity. The different adjustments made in different studies could potentially affect the meta-analysis results. That might be the case with the analysis presented in Figure 2, which shows significant heterogeneity between the analyzed studies. 

Secondly, the robustness of some of the analyses we performed was affected by the way in which measurements of retinal vascular diameter were stratified. When retinal vascular diameter was examined as a categorical variable, some studies divided their measurements into tertiles, some into quartiles, and some into quintiles. Thus, we had to disqualify some of the studies that could otherwise potentially have been included in the meta-analysis [25,27].

Thirdly, while most of the studies reported narrower CRAE and wider CRVE, some reported the opposite [3,8,16,39], and one study reported a U-shaped association of CRAE with kidney dysfunction [20]. All of those studies were removed from the meta-analysis. To obtain more accurate observations, retinal vascular diameter should preferably be studied separately, for narrowing and for widening of both CRAE and CRVE. 

Fourth, the definition of kidney dysfunction varied between different studies. In some of the reports CKD, ESRD, and albuminuria were defined using classifications which are not updated according to the current standards. For example, Lim et al. defined albuminuria as an albumin to creatinine ratio (ACR) greater than 17 mg/g in women and an ACR greater than 25 mg/g in men [16], a classification that does not exist today. In others, a general definition termed “renal dysfunction” was used, and included non-official criteria [2]. In such cases, it was difficult to compare their findings with other studies, let alone include them in the meta-analysis, even if their results were statistically significant. Furthermore, most reports did not measure the protein to creatinine ratio (PCR), which is more reliable than ACR in the prediction of kidney disease progression.

## 5. Conclusions and Further Study

The findings of our study unequivocally confirmed that retinal vascular signs are associated with kidney diseases. Both in diabetic and in nondiabetic patients, retinopathy was found to correlate with CKD and ESRD. 

The abundance of evidence attesting to an association of retinal vascular diameter with findings of early kidney dysfunction suggests that fundus photography might serve as a valuable screening test for patients at risk of developing CKD. Currently lacking, however, is adequate evidence for an association of retinal vascular diameter with kidney-related morbidity and mortality. 

In addition, the retinal vascular architecture should be studied as a whole, using artificial intelligence, machine learning, and deep learning models, and taking into account the additive effects of multiple signs and their association with kidney disease. Finally, future works should stratify their study populations by potential confounders such as age, gender, obesity, hypertension, ethnicity, and others.

## Figures and Tables

**Table 1:** Retinal microvascular and incident CKD.

S/N	Author	Sample Size	Demographics		Main Results	HR (95% CI)	Adjustment Parameters	Follow-Up (Years)
			Age (Years)	% Males				
Retinal vascular diameter								
1	Yip (2017) [6]	*n*= 1256	53.6–62.4 **	36.9–57.7	CRAE and CRVE were associated with incident CKD	1.34 (1.0–1.78) for CRAE (per SD ↓) and 2.35 (1.12–5.94) for CRVE (per SD ↑)	Age, sex, education level, eGFR, glucose levels, SBP, hypertension, smoking, anti-hypertensive medications, hsCRP, total cholesterol, HDL, fellow retinal vessel caliber (CRAE in models including CRVE and vice versa)	6 *
2	Yau (2011) [9]	*n* = 4594	59–69 **	40.1–49.1	CRAE was associated with incident CKD in whites	1.78 (1.01–3.15) for CRAE in tertile 1	Age, sex, study center, venular caliber, SBP, DM, medication for diabetes, antihypertensive medications, BMI, triglycerides, smoking, fasting glucose level, CRP, HbA1c level, logACR, any retinopathy	4.8 *
3	Sabanayagam (2011) [25]	N = 3302	54.1–71.7 **	26–48.8	Retinal vascular diameter was not associated with incident CKD	-	Age, sex, diabetes, hypertension, education, smoking, alcohol intake, BMI, diabetes, glycated hemoglobin, CRP, total cholesterol and HDL, cholesterol	15 *
Retinopathy								
1	Yip (2017) [6]	*n* = 1256	53.6–62.4 **	36.9–57.7	Retinopathy was associated with incident CKD	2.54 (1.48–4.36)	Age, sex, education level, baseline eGFR, glucose levels, SBP, hypertension, smoking, anti-hypertensive medications, hsCRP, total cholesterol, HDL cholesterol	6 *

* mean/median; ** ranges of means/medians between different study groups. Abbreviations: HR: hazard ratio; CI: confidence interval; SD: standard deviation; CRAE: central retinal artery equivalent; CRVE: central retinal venous equivalent; CKD: chronic kidney disease; eGFR: estimated glomerular filtration rate; ACR: albumin creatinine ratio; SBP: systolic blood pressure; hsCRP: high-sensitivity C-reactive protein; HDL: high-density lipoprotein; DM: diabetes mellitus; HbA1c: glycosylated hemoglobin; BMI: body mass index.

**Table 2:** Retinal microvascular signs and incident ESRD.

S/N	Author	Sample Size	Demographics		Main Results	HR (95% CI)	Adjustment Parameters	Follow-Up (Years)
			Age (Years)	% Males				
Retinal vascular diameter								
1	Baumann (2014) [26]	*n* = 164	60.8 *	57	CRAE and CRAE + albuminuria were associated with incident ESRD	3.0 (1.2–7.5) for CRAE (in tertile 1) and 10.0 (2.6–38.7) for CRAE + albuminuria	Age, eGFR, DM, SBP, RAAS inhibition	3.8 *
2	Grunwald (2014) [12]	*n* = 1852	62 *	54.1	AVR was associated with incident ESRD	3.11 (1.51–6.4) for AVR in quartile 4. 1.32 (1.03–1.7) for AVR Per SD ↑	Age, SBP, race, DM, BMI, smoking, clinical site, 24 h urine protein, eGFR	2.3 *
3	Yip (2015) [8]	*n* = 5763	55.1 *	48.7	Retinal vascular diameter was not associated with incident ESRD	-	Age, gender, race, hypertension, DM, eGFR	4.3 *
Retinopathy								
1	Grunwald (2014) [12]	*n* = 1852	62 *	54.1	Retinopathy was not associated with incident ESRD in the fully adjusted model	1.26 (0.76–2.11)	Age, SBP, race, DM, BMI, smoking, clinical site, 24 h urine protein, eGFR	2.3 *
2	Yip (2015) [8]	*n* = 5763	55.1 *	48.7	Retinopathy was associated with incident ESRD	2.51 (1.14–5.54)	Age, gender, race, hypertension, DM, eGFR	4.3 *

* median. Abbreviations: HR: hazard ratio; CI: confidence interval; SD: standard deviation; CRAE: central retinal artery equivalent; CRVE: central retinal venous equivalent; AVR: arterio-venous ratio; ESRD: end-stage renal disease; eGFR: estimated glomerular filtration rate; SBP: systolic blood pressure; DM: diabetes mellitus; BMI: body mass index; RAAS: renin angiotensin aldosterone system.

**Table 3:** Retinal microvascular signs and prevalent CKD.

S/N	Author	Sample Size	Demographics		Main Results	OR (95% CI)	Adjustment Parameters	Follow-Up (Years)
			Age (Years)	% Males				
Retinal vascular diameter								
1	Bao (2015) [3]	*n* = 5925	59.1 *	45.3	AVR was associated with prevalent CKD	1.24 (1.0–1.53) for AVR in quartile 1	Age, sex, smoking, alcohol consumption, BMI, education, total cholesterol, triglycerides, LDL and HDL levels	1–2 *
2	Sabanayagam (2008) [15]	*n* = 3280	56.4–58.5 **	39.6–57.7	CRAE was associated with prevalent CKD	1.42 (1.03–1.96) for CRAE in quartile 1; 1.11 (1.0–1.24) for CRAE per SD ↓	Age, gender, smoking, DM, hypertension, BMI, total cholesterol, HDL levels	1.8 *
3	Sabanayagam (2009) [14]	*n* = 3602	45.2–54.7 **	44.4–51.7	CRAE was associated with prevalent CKD	1.68 (1.04–2.71) for CRAE in quartile 1; 1.2 (1.02–1.4) for CRAE per SD ↓; 3.61 (1.86–6.93) for CRAE + hypertension	Age, sex, ethnicity, education, smoking, alcohol consumption, DM, mean arterial blood pressure, BMI, total cholesterol, HDL cholesterol	4 *
4	Liew (2012) [27]	*n* = 2971	59.1–71.2 **	-	CRVE was associated with prevalent CKD	1.2 (1.0–1.5) for CRVE in quintile 5	Age, gender, fasting plasma glucose, SBP	–
5	Lim (2013) [16]	*n* = 3280	56.8–66.1 **	-	CRAE and CRVE were not associated with prevalent CKD	-	Age, sex, hypertension, DM, smoking, history of stroke, BMI, lipids, education	1–3 *
6	Phan (2016) [28]	*n* = 1512	60–69.4 **	65.1–77.3	CRAE and CRVE were not associated with prevalent CKD	-	Age, sex, BMI, ethnicity, hypertension, DM, cholesterol level	3.5 *
7	Gu (2015) [29]	*n* = 292	65.7–72 **	45–53.7	CRAE < 150 μm discriminated CKD stage 1 from stage 2 and beyond	2.81 (1.68–4.69)	Sex, age, DBP, smoking	4 *
8	Ooi (2011) [30]	*n* = 252	60.5–61 **	65.1	CRAE and CRVE discriminated CKD stage 1–2 from stage 3–5	2.84 (1.25–6.46) for CRAE in quartile 1; 4.75 (2.00–11.3) for CRVE in quartile 1	Age, gender, hypertension, DM, dyslipidemia, smoking history	
Retinopathy								
1	Bao (2015) [3]	*n* = 5925	59.1 **	45.3	Retinopathy was associated with prevalent CKD	1.34 (1.07–1.68)	Age, sex, smoking, alcohol consumption, BMI, education, total cholesterol, triglycerides, LDL and HDL levels	1–2 *
2	Liew (2012) [27]	*n* = 2971	59.1–71.2 **	-	Retinopathy was associated with prevalent CKD	1.2 (1.0–1.5)	Age, gender, fasting plasma glucose, SBP	-
3	Sabanayagam (2008) [15]	*n* = 3280	56.4–58.5 **	39.6–57.7	Retinopathy was associated with prevalent CKD	1.56 (1.14–2.14)	Age, gender, smoking, DM, hypertension, BMI, total cholesterol, HDL levels	1.8 *
4	Wong (2004) [2]	*n* = 10,056	59.7–61.8 **	43.9–58.5	Any retinopathy, microaneurysms, retinal hemorrhages, and soft exudates were associated with renal dysfunction **	2.0 (1.4–2.8) for any retinopathy; 2.0 (1.3–3.1) for microaneurysms; 2.6 (1.6–4.0) for retinal hemorrhages; 2.7 (1.6–4.8) for soft exudates	Age, gender, race, field center, DM, fasting glucose, antihypertensive medication, MABP, fasting HDL cholesterol and triglyceride, BMI, smoking, alcohol consumption	6 *
Other retinal microvascular signs								
1	Liew (2012) [27]	*n* = 2971	59.1–71.2 **	-	Retinal microvascular signs were not associated with prevalent CKD	-	Age, gender, fasting plasma glucose, SBP	-
2	Lim (2013) [16]	*n* = 3280	56.8–66.1 **	-	Retinal microvascular signs were not associated with prevalent CKD	-	Age, sex, hypertension, DM, smoking, history of stroke, BMI, lipids, education	1–3 *
3	Sng (2010) [31]	*n* = 884	60.4 *	55.3	Df was associated with prevalent CKD	2.1 (1.15–3.83) for Df in quintile 1	Age, gender, ethnicity, DM, SBP, BMI, alcohol consumption, smoking status, total cholesterol, HDL cholesterol, DR, DR treatment	-
4	Wong (2004) [2]	*n* = 10,056	59.7–61.8 **	43.9–58.5	AVN was associated with renal dysfunction ***	1.4 (1.0–1.9)	Age, gender, race, field center, DM, fasting glucose, antihypertensive medication, MABP, fasting HDL cholesterol and triglyceride, BMI, smoking, alcohol consumption	6 *
5	Vadala (2018) [13]	*n* = 120	50.5 *	68.3	Decreased retinal and choroidal thickness, superficial and deep parafoveal vascular density, were associated with prevalent CKD	*p* < 0.05 for each of the parameters examined **	-	1 *

* mean/median; ** ranges of means/medians between different study groups; *** defined as 0.4 mg/dL or more increase in Cr, death/hospitalization for renal disease. Abbreviations: OR: odds ratio; CI: confidence interval; SD: standard deviation; CRAE: central retinal artery equivalent; CRVE: central retinal venous equivalent; eGFR: estimated glomerular filtration rate; SBP: systolic blood pressure; DBP: diastolic blood pressure; DM: diabetes mellitus; BMI: body mass index; MABP: mean arterial blood pressure; HDL: high-density lipoprotein; LDL: low-density lipoprotein; DR: diabetic retinopathy; CKD: chronic kidney disease; AVN: arterio-venous nicking; Df: fractal dimension; FAN: focal arteriolar narrowing; Cr: creatinine.

**Table 4:** Retinal microvascular signs and prevalent ESRD.

S/N	Author	Sample Size	Demographics		Main Results	OR (95% CI)	Adjustment Parameters	Follow-Up (Years)
			Age (Years)	% Males				
1	Yip (2015) [8]	*n* = 5763	55.1 *	48.7	Retinopathy was associated with prevalent ESRD	3.21 (1.28, 8.05)	Age, gender, race	4.3 *
2	Grunwald (2019) [32]	*n* = 1583	60 *	54.1	Changes in CRAE or CRVE, and retinopathy worsening, were not associated with prevalent ESRD or 50% decline in eGFR between initial and follow-up photography	-	eGFR, level of retinopathy, age, race, BMI, smoking, DM, SBP, clinical site (adjustment for retinopathy); eGFR, retinal vascular diameter, age, race, BMI, smoking, DM, SBP, clinical site (adjustment for retinal vascular diameter)	3.5 *

* mean; Abbreviations: OR: odds ratio; CI: confidence interval; CRAE: central retinal artery equivalent; CRVE: central retinal venous equivalent; eGFR: estimated glomerular filtration rate; SBP: systolic blood pressure; DM: diabetes mellitus; BMI: body mass index; HDL: high-density lipoprotein; Df: fractal dimension; ESRD: end-stage renal disease.

**Table 5:** Retinal microvascular signs and eGFR.

S/N	Author	Sample Size	Demographics		Main Results	OR (95% CI)/β (95% CI/±SE)	Adjustment Parameters	Follow-Up (Years)
			Age (Years)	% Males				
Retinal vascular diameter								
1	Lim (2013) [16]	*n* = 3280	56.8–66.1 **	-	CRAE was associated with lower eGFR	β 0.77 (0.20–1.35) for CRAE (per SD ↑)	Age, sex, hypertension, DM, smoking, history of stroke, BMI, lipids, education	1–3 *
2	Baumann (2009) [33]	*n* = 67	61.5–62.3 **	50–61.5	CRAE was associated with eGFR decline	CRAE independently predicted eGFR (β = 0.33)	-	-
3	Gu (2015) [29]	*n* = 292	65.7–72 **	45–53.7	CRAE was associated with eGFR decline	β 2.27 (0.53–4.01) for CRAE per SD ↓	Sex, age, DBP, smoking	4 *
Retinopathy								
1	Edwards (2005) [34]	*n* = 1394	78 *	38.9	Any retinopathy, retinal hemorrhages, microaneurysms and hard/soft exudates were associated with 20% or more decline in eGFR	OR 2.84 (1.56–5.16) for any retinopathy; OR 2.18 (1.01–4.71) for retinal hemorrhages; OR 2.25 (1.0–5.09) for microaneurysms; OR 6.63 (2.7–16.3) for hard/soft exudates	Age, race, sex, weight, DM, hypertension, ACE inhibitor use, proteinuria	5 *
2	Grunwald (2019) [32]	*n* = 1583	60 *	54.1	Retinopathy worsening was not associated with change in eGFR slope	-	eGFR, retinopathy level, age, race/ethnicity, BMI, smoking	3.5 *
3	Hwang (2016) [35]	*n* = 523	63.9–73.2 **	48.5–57.6	Patients with retinopathy showed a faster eGFR decline than those without retinopathy	−7.2 ± 10.2 vs. −3.1 ± 10.1	Age, BMI, DM, smoking, previous CV events, serum albumin level	2–2.7 *
Other retinal microvascular signs								
1	Edwards (2005) [34]	*n* = 1394	78 *	38.9	AVN and FAN were not associated with change in eGFR	-	Age, race, sex, weight, DM, hypertension, ACE inhibitor use, proteinuria	5 *
2	Lim (2013) [16]	*n* = 3280	56.8–66.1 **	-	Df and AVN were associated with lower eGFR	β 0.83 (0.17–1.48) for Df; β −3.09 (−5.25–(−0.94)) for AVN	Age, sex, hypertension, DM, smoking, history of stroke, BMI, lipids, education	1–3 *

* mean; ** ranges of means/medians between different study groups. Abbreviations: OR: odds ratio; CI: confidence interval; SE: standard error; SD: standard deviation; CRAE: central retinal artery equivalent; CRVE: central retinal venous equivalent; eGFR: estimated glomerular filtration rate; DBP: diastolic blood pressure; DM: diabetes mellitus; BMI: body mass index; Df: fractal dimension; AVN: arterio-venous nicking; FAN: focal arteriolar narrowing; ACE: angiotensin-converting enzyme; CV: cardiovascular.

**Table 6:** Retinal microvascular signs and albuminuria.

S/N	Author	Sample Size	Demographics		Main Results	OR (95% CI)/β (95% CI)	Adjustment Parameters	Follow-Up (Years)
			Age (Years)	% Males				
Retinal vascular diameter								
1	Bao (2015) [3]	*n* = 5925	59.1 *	45.3	AVR was associated with albuminuria	OR 1.26 (1.01–1.56) for AVR in quartile 1	Age, sex, smoking, alcohol consumption, BMI, education, total cholesterol, triglycerides, LDL and HDL levels	1–2 *
2	Awua-larbi (2011) [20]	*n* = 5897	63.2 *	47.9	CRAE was associated with albuminuria	OR 1.55 (1.17–2.04) for CRAE in quintile 1; OR 1.44 (1.07–1.93) for CRAE in quintile 5	Age, sex, race, SBP, antihypertensive drugs, use of RAAS inhibitor, DM, smoking, waist circumference, education, access to healthcare	-
3	Garcia-Ortiz (2012) [36]	*n* = 205	55.6 *	57.1	CRVE and AVR were associated with albuminuria	β −27.350 (−53.66–(− 1.039)) for AVR; β 0.18 (0.04–0.32) for CRVE	Sex, age	2 *
4	Lim (2013) [16]	*n* = 3280	56.8–66.1 **	−	Retinal vascular diameter was not associated with albuminuria	-	Age, sex, hypertension, DM, smoking, history of stroke, BMI, lipids, education	3 *
5	Sabanayagam (2008) [15]	*n* = 3280	56.4–58.5 **	39.6–57.7	CRAE and CRVE were associated with albuminuria	OR 1.80 (1.11–2.91) for CRAE in quartile 1 and OR 1.22 (1.03–1.44) for CRAE per SD ↓; OR 1.63 (1.02–2.60) for CRVE in quartile 2	Age, gender, smoking, DM, hypertension, BMI, total cholesterol, HDL levels	1.8 *
Retinopathy								
1	Bao (2015) [3]	*n* = 5925	59.1 *	45.3	Retinopathy was associated with albuminuria	OR 1.34 (1.06–1.68)	Age, sex, smoking, alcohol consumption, BMI, education, total cholesterol, triglycerides, LDL and HDL levels	1–2 *
2	Sabanayagam (2008) [15]	*n* = 3280	56.4–58.5 **	39.6–57.7	Retinopathy was associated with micro/macroalbuminuria	OR 1.88 (1.13–3.15)	Age, gender, smoking, DM, hypertension, BMI, total cholesterol, HDL levels	1.8 *
Other retinal microvascular signs								
1	Lim (2013) [16]	*n* = 3280	56.8–66.1 **	-	Df, FAN, and AVN were associated with albuminuria	β −0.21 (−0.32–(−0.10)) for Df (per SD ↑); β 0.60 (0.28–0.92) for FAN; β 0.47 (0.12–0.83) for AVN	Age, sex, hypertension, DM, smoking, history of stroke, BMI, lipids, education	3 *

* mean/median; ** ranges of means/medians between different study groups. Abbreviations: OR: odds ratio; CI: confidence interval; SD: standard deviation; CRAE: central retinal artery equivalent; CRVE: central retinal venous equivalent; SBP: systolic blood pressure; DM: diabetes mellitus; BMI: body mass index; Df: fractal dimension; AVN: arterio-venous nicking; FAN: focal arteriolar narrowing; HDL: high-density lipoprotein; LDL: low density lipoprotein; RAAS: renin angiotensin aldosterone system.

**Table 7:** Retinal microvascular signs and incident ESRD in diabetic patients.

S/N	Author	Sample Size	Demographics		Main Results	HR (95% CI)	Adjustment Parameters	Follow-Up (Years)
			Age (Years)	% Males				
Retinal vascular diameter								
1	Yip (2015) [8]	*n* = 5763	55.1 *	48.7	CRAE and CRVE were not associated with incident ESRD	-	Age, gender, race, hypertension, eGFR, HbA1c	4.3 *
Retinopathy								
1	Yip (2015) [8]	*n* = 5763	55.1 *	48.7	Retinopathy was associated with incident ESRD	2.6 (1.01–6.66)	Age, gender, race, hypertension, eGFR, HbA1c	4.3 *
2	Lee (2014) [38]	*n* = 51	58.2 *	60.8	Nonperfusion area ≥ 10 disc areas (on FA) was associated with incident ESRD	6.64 (1.96–22.52)	-	2 *
Other retinal microvascular signs								
1	Yip (2015) [8]	*n* = 5763	55.1 *	48.7	Df was not associated with incident ESRD	-	Age, gender, race, hypertension, eGFR, HbA1c	4.3 *

* mean; Abbreviations: HR: hazard ratio; CI: confidence interval; SD: standard deviation; CRAE: central retinal artery equivalent; CRVE: central retinal venous equivalent; Df: fractal dimension; FA: fluorescein angiography; ESRD: end-stage renal disease; HbA1c: glycosylated hemoglobin.

**Table 8:** Retinal microvascular signs and prevalent CKD in diabetic patients.

S/N	Author	Sample Size	Demographics		Main Results	OR (95% CI)	Adjustment Parameters	Follow-Up (Years)
			Age (Years)	% Males				
Retinal vascular diameter								
1	Bao (2015) [3]	*n* = 5925	59.1 *	45.3	CRAE, CRVE, and AVR were not associated with prevalent CKD	-	Age, sex, smoking, alcohol consumption, BMI, education, total cholesterol, triglycerides, LDL and HDL levels	1–2 *
2	Liew (2012) [27]	*n* = 2971	59.1–71.2 **	-	CRVE was not associated with prevalent CKD	1.2 (0.6–2.4) for CRVE in quintile 5	Age, gender, fasting plasma glucose, SBP	-
3	Mckay (2018) [39]	*n* = 1072	63 *	51	CRAE, CRVE, and AVR were not associated with reduced renal function ***	-	Age, gender, SBP, HbA1c	3 *
Retinopathy								
1	Sabanayagam (2008) [8]	*n* = 3280	56.4–58.5 **	39.6–57.7	Retinopathy was associated with prevalent CKD	1.50 (1.00–2.25)	Age, gender, smoking, DM, hypertension, BMI, total cholesterol, HDL cholesterol	1.8 *
2	Liew (2011) [27]	*n* = 2971	59.1–71.2 **		Retinopathy was associated with prevalent CKD	1.3 (0.7–2.5)	Age, gender, fasting plasma glucose, SBP	-
3	Bao (2015) [3]	*n* = 5925	59.1 *	45.3	Retinopathy was associated with prevalent CKD	1.63 (0.35–7.58)	Age, sex, smoking, alcohol consumption, BMI, education, total cholesterol, triglycerides, LDL and HDL levels	1–2 *
4	Zhang (2014) [17]	*n* = 523	36–59.7 **	54–61	NPDR and PDR were associated with prevalent CKD	2.22 (1.01–4.86) for NPDR; 3.52 (1.3–9.55) for PDR	Age, gender, SBP, hypertension, HbA1c, duration of diabetes	-
5	Mottl (2020) [19]	*n* = 1292	60.4–72 **	40–51	Retinopathy was associated with prevalent CKD in subgroups of the study population	2.7 (1.2–6.1) for non-Hispanic blacks; 2.6 (1.3–5.5) for obesity; 2.5 (1.1–5.7) for patients not using RAAS blockers	Age, gender, HbA1c, SBP, DBP	-
6	Wong (2004) [37]	*n* = 10,056	59.7–61.8 **	43.9–58.5	Retinopathy was associated with renal dysfunction †	2.6 (1.6–4.3) for diabetic patients; 2.1 (1.2–3.8) for diabetes + hypertension	Age, gender, race, field center, DM, fasting glucose, antihypertensive medication, MABP, fasting HDL cholesterol, triglyceride, BMI, smoking, alcohol consumption	6 *
Other retinal microvascular signs								
1	Mckay (2018) [39]	*n* = 1072	63 *	51	Df and tortuosity were not associated with reduced renal function	-	Age, gender, SBP, HbA1c	3 *

* mean/median; ** ranges of means/medians between different study groups; *** defined as eGFR of < 60 mL/min/1.73 m^2^ at follow-up or a reduction in eGFR of at least 15% between baseline and follow-up; † defined as increase in serum creatinine levels (of at least 0.4 mg/dL) or a hospitalization discharge or death coded for renal disease over the 6 y period between the second and fourth examinations. hospitalization discharge or death included the diagnosis of chronic renal disease. Abbreviations: OR: odds ratio; CI: confidence interval; CRAE: central retinal artery equivalent; CRVE: central retinal venous equivalent; AVR: arterio-venous ratio; Df: fractal dimension; DM: diabetes mellitus; BMI: body mass index; HDL: high-density lipoprotein; LDL: low-density lipoprotein; HbA1c: glycosylated hemoglobin; SBP: systolic blood pressure; DBP: diastolic blood pressure; MABP: mean arterial blood pressure; RAAS: renin angiotensin aldosterone system; CKD: chronic kidney disease; PDR: proliferative diabetic retinopathy; NPDR: non-proliferative diabetic retinopathy.

**Table 9:** Retinal microvascular signs and DN.

S/N	Author	Sample Size	Demographics		Main Results	OR (95% CI)	Adjustment Parameters	Follow-Up (Years)
			Age (Years)	% Males				
Retinal vascular diameter								
1	Broe (2014) [18]	*n* = 185	21 *	-	CRAE and CRVE were associated with DN	2.63 (1.09–6.36) per 10 µm ↓ for CRAE; 1.76 (1.05–2.94) per 10 µm ↑ for CRVE	-	16 *
Other retinal microvascular signs								
1	Broe (2014) [40]	*n* = 180	-	-	Df was associated with DN	1.40 (1.10–1.79) per 0.01 ↓ in Df	Sex, age, duration of diabetes, HbA1c, SBP, DBP, BMI, retinopathy, VPT, albuminuria, CRAE, CRVE	16 *
2	Rasmussen (2017) [41]	*n* = 181	37 *	50.8	Arteriolar BC was associated with DN	3.1 (1.01–9.54)	Sex, age, duration of diabetes, SBP, DBP, HbA1c, retinopathy, VPT	-

* mean; Abbreviations: OR: odds ratio; CI: confidence interval; µm: micrometers; CRAE: central retinal artery equivalent; CRVE: central retinal venous equivalent; Df: fractal dimension; BC: branching coefficient; LDR: length to diameter ratio; DN: diabetic nephropathy; BMI: body mass index; HbA1c: glycosylated hemoglobin; SBP: systolic blood pressure; DBP: diastolic blood pressure; VPT: vibration perception threshold.

**Table 10:** Retinal microvascular signs and concurrent eGFR in diabetic patients.

S/N	Author	Sample Size	Demographics		Main Results	OR (95% CI)/β (95% CI)	Adjustment Parameters	Follow-Up (Years)
			Age (Years)	% Males				
Retinal vascular diameter								
1	Edwards (2005) [34]	*n* = 1394	78 *	38.9	AVR was not associated with 20% decline in eGFR	-	Cr, age, sex, race, weight, BP, ACE-inhibitor use, albuminuria/proteinuria	9 *
2	Mckay (2018) [39]	*n* = 1072	63 *	51	CRAE, CRVE, and AVR were not associated with eGFR decline at follow-up	-	Age, gender, SBP, HbA1c	3 *
Retinopathy								
1	Edwards (2005) [34]	*n* = 1394	78 *	38.9	Microaneurysms and hard/soft exudates were associated with 20% or more decline in eGFR	OR 4.1 (1.04–16.3) for microaneurysms; OR 7.09 (1.1–45.6) for hard/soft exudates	Cr, age, sex, race, weight, BP, ACE-inhibitor use, albuminuria/proteinuria	9 *
2	Grunwald (2019) [32]	*n* = 1583	60 *	54.1	eGFR slope between patients with retinopathy worsening did not differ from eGFR slope in patients without retinopathy worsening	-	eGFR, retinopathy, age, race, BMI, smoking	3.5 *
Other retinal microvascular signs								
1	Edwards (2005) [34]	*n* = 1394	78 *	38.9	AVN and FAN were not associated with 20% decline in eGFR	-	Cr, age, sex, race, weight, BP, ACE-inhibitor use, albuminuria/proteinuria	9 *
2	Mckay (2018) [39]	*n* = 1072	63 *	51	Df and tortuosity were not associated with eGFR decline at follow-up	-	Age, gender, SBP, HbA1c	3 *

* mean; Abbreviations: OR: odds ratio; CI: confidence interval; CRAE: central retinal artery equivalent; CRVE: central retinal venous equivalent; AVR: arterio-venous ratio; Df: fractal dimension; AVN: arterio-venous nicking; FAN: focal arteriolar narrowing; BMI: body mass index; HbA1c: glycosylated hemoglobin; BP: blood pressure; SBP: systolic blood pressure; ACE: angiotensin-converting enzyme; eGFR: estimated glomerular filtration rate; Cr: creatinine.

**Table 11:** Retinal microvascular signs and concurrent albuminuria in diabetic patients.

S/N	Author	Sample Size	Demographics		Main Results	OR (95% CI)	Adjustment Parameters	Follow-Up (Years)
			Age (Years)	% Males				
Retinal vascular diameter								
1	Benitez (2018) [42]	*n* = 963	14.4 *	53	exMWa was associated with high risk for albuminuria	1.67 (1.17–2.38) for exMWa; 1.39 (0.98–1.99) for exMWv	BMI SDS, duration, and SBP SDS	-
2	Grauslund (2009) [44]	*n* = 208	57.9 *	-	CRAE and AVR were associated with albuminuria	2.17 (1.29–3.68) for CRAE per SD ↓; 1.48 (1.01–2.16) for AVR per SD ↓	Age, sex, duration of diabetes, HbA1c, SBP, pack years	-
3	Awua-larbi (2011) [20]	*n* = 5897	63.2 *	47.9	CRAE was associated with albuminuria	2.26 (1.34–3.81) for CRAE in quintile 1	Age, sex, race, SBP, antihypertensive medication, RAAS inhibitors, smoking, waist circumference, education, access to healthcare	-
4	Keel (2017) [21]	*n* = 483	14.5 *	53	CRAE, CRVE, and AVR were not associated with albuminuria	-	Age, sex, ethnicity, HbA1c, total and LDL cholesterol, BMI, duration of diabetes, SBP	-
5	Bao (2015) [3]	*n* = 5925	59.1 *	45.3	CRAE was associated with albuminuria	2.17 (1.03–4.56) for CRAE in quartile 2	Age, sex, BMI, education, hypertension, diabetes, smoking, drinking, total cholesterol, triglyceride, LDL, HDL	1–2 *
Retinopathy								
1	Mottl (2012) [19]	*n* = 1292	60.4–72 **	40–51	Any retinopathy and moderate-to-severe retinopathy were associated with micro/macro-albuminuria	1.8 (1.1–2.8) for any retinopathy; 2.7 (1.4–5.5) for moderate-severe retinopathy	-	-
2	Sabanayagam (2008) [15]	*n* = 3280	56.4–58.5 **	39.6–57.7	Retinopathy was not associated with albuminuria	-	Age, gender, smoking, diabetes, hypertension, BMI, total cholesterol, HDL cholesterol	1.8 *
3	Bao (2015) [3]	*n* = 5925	59.1 *	45.3	Retinopathy was not associated with albuminuria	-	Age, sex, BMI, education, hypertension, diabetes, smoking, drinking, total cholesterol, triglyceride, LDL, HDL	1–2 *
4	Ha M (2019) [45]	*n* = 103	61–67.3 **	20–31	Vitreous hemorrhage showed a higher incidence in the microalbuminuria group and in the advanced nephropathy group than in the no nephropathy group	*p* = 0.017 (this study was retrospective)	-	-
Other retinal microvascular signs								
1	Grauslund (2010) [46]	*n* = 208	57.8 *	46.7–62.4	Df was not associated with albuminuria	-	Age, gender, duration of diabetes, SBP, smoking	-
2	Benitez (2012) [43]	*n* = 666	13.5 *	47–54	LDRv and STv were associated with increased AER	1.69 (1.17–2.44) for LDRv in quartile 4; 1.55 (1.08–2.22) for STv in quartile 1	Age, diabetes duration, HbA1c, BP, BMI, cholesterol	3.7 **
3	Sasongko (2012) [47]	*n* = 944	13.6–14 **	44.1–49.8	Arteriolar tortuosity index was associated with increased AER	1.56 (1.06–2.28) for arteriolar tortuosity index per SD ↑	Age, sex, duration of diabetes, HbA1c, SBP, cholesterol, BMI, retinal vessel diameter	-
4	Cankurtaran (2019) [5]	*n* = 137	54.8–56.7 **	45.4–54.7	SCP, DCP vessel densities, whole disc, and peripapillary areas in microalbuminuric patients differed significantly from those in normoalbuminuric or control patients	*p* < 0.05 in all comparisons (multilinear regression analysis was not performed)	-	-
5	Garrido (2019) [48]	*n* = 21	49.76 *	-	CMT and CT were associated with albuminuria	*p* < 0.05 in all comparisons (only bivariate analysis was performed)	-	-

* mean/median; ** ranges of means/medians between different study groups. Abbreviations: OR: odds ratio; CI: confidence interval; SD: standard deviation; CRAE: central retinal artery equivalent; CRVE: central retinal venous equivalent; AVR: arterio-venous ratio; exMWa: extended mean arteriolar width; exMWv: extended mean venular width; Df: fractal dimension; LDRv: venular length to diameter ratio; STv: venular simple tortuosity; AER: albumin excretion rate; SCP: superficial capillary plexus; DCP: deep capillary plexus; CMT: central macular thickness; CT: choroidal thickness; BMI: body mass index; HbA1c: glycosylated hemoglobin; BP: blood pressure; SBP: systolic blood pressure; SDS: standard deviation score; BP: blood pressure; LDL: low-density lipoprotein; HDL: high-density lipoprotein; RAAS: renin angiotensin aldosterone system.

## Supplementary Materials

The following are available online at https://www.mdpi.com/article/10.3390/jpm11070665/s1, Study protocol, Table S1: Summary of bias findings using the QUIPS tool.

## Appendix A

Search strategy and study selection

Searches were carried out in PubMed and Embase, for studies published until 1 January 2020, using the following combination of search terms:

Pubmed (1331)

(((((((((((((((Renal Insufficiency[MeSH Terms]) OR Renal Insufficiency) OR “Renal Insufficiency”)) OR ((Renal Failure) OR “Renal Failure”)) OR ((Kidney Failure) OR “Kidney Failure”)) OR Kidney Diseases[MeSH Terms]) OR ((((Diabetic Nephropathies[MeSH Terms]) OR Diabetic Nephropathies) OR Diabetic Nephropathy) OR Nephropathy)) OR ((“Renal”) OR “Kidney”)) OR (((((Glomerular filtration rate) OR “Glomerular filtration rate”) OR Estimated glomerular filtration rate) OR “GFR”) OR “eGFR”)) OR ((“CKD”) OR “CRF”))) AND ((((((((((((((Retinal Vascular Diameter) OR Retinal Vascular Caliber) OR “Retinal Vascular Caliber”) OR CRAE) OR CRVE) OR Retinopathy) OR Retinal Arteriovenous Ratio) OR AVR)) OR ((((((Tortuosity) OR Arteriovenous Nicking) OR Fractal Dimension) OR Optimality Ratio) OR Branching Angle) OR Tortuosity))))) AND ((“Retina”) OR “Retinal”))) AND (“1 January 1950”[Date-Publication]: “1 January 2020”[Date-Publication]))) NOT (((((((((((((((((((case reports[Publication Type]) OR clinical conference[Publication Type]) OR Consensus Development Conference[Publication Type]) OR Consensus Development Conference, NIH[Publication Type]) OR Dataset[Publication Type]) OR English Abstract[Publication Type]) OR Guideline[Publication Type]) OR Historical Article[Publication Type]) OR Introductory Journal Article[Publication Type]) OR Meta-Analysis[Publication Type]) OR Review[Publication Type]) OR News[Publication Type]) OR Newspaper Article[Publication Type]) OR Practice Guideline[Publication Type]) OR Research Support, N I H, Extramural[Publication Type]) OR Scientific Integrity Review[Publication Type]) OR Technical Report[Publication Type]) OR Twin Study[Publication Type]) OR Video-Audio Media[Publication Type]) NOT (“Animals”[Mesh] NOT (“Animals”[Mesh] AND “Humans”[Mesh]))

Embase (534)

(‘retina’/exp OR ‘retinal vascular disease’/kw OR ‘retinal vascular caliber’/kw OR ‘retinal vascular tortuosity’/kw OR ‘retina blood vessel’/exp) AND (‘kidney failure’/exp OR ‘glomerulus filtration rate’/kw OR ‘albuminuria’/kw OR ‘kidney disease’/exp OR ‘diabetic nephropathy’/kw OR ‘albumin creatinine ratio’/kw) AND [english]/lim AND [article]/lim AND [<1966–2020]/py.
