# Supplementary material for: Retinal Vascular Signs as Screening and Prognostic Factors for Chronic Kidney Disease: A Systematic Review and Meta-Analysis of Current Evidence
Source: J Pers Med. 2021 Jul 15;11(7):665. doi: 10.3390/jpm11070665 (PMC8307097; doi:10.3390/jpm11070665)
Supplement: Supplementary file 1 [file jpm-11-00665-s001.zip › jpm-1286843-supplementary.pdf]

Table S1. Summary of bias findings using the QUIPS tool							
**Author**	**Study participation**	**Study attrition**	**Prognostic factor measurement**	**Outcome measurement**	**Study confounding**	**Statistical analysis and reporting**	**Overall risk of bias**
Baumann (2014)	Low	Moderate	Low	Low	Moderate	Low	**Low**
Benitez (2012)	Low	High	Moderate	Low	Moderate	Low	**Moderate**
Grunwald (2014)	Low	Low	Low	Low	Moderate	Low	**Low**
Sabanayagam (2011)	Low	Low	Low	Low	Low	Low	**Low**
Wong (2003)	Low	High	Low	Low	Low	Low	**Moderate**
Yau (2011)	Low	Unknown	Low	Low	Low	Low	**Low**
Yip (2015)	Low	Low	Low	Moderate	High	Low	**Moderate**
Yip (2017)	Low	High	Low	Low	Low	Low	**Moderate**
Awua-larbi (2011)	Low	High	Low	Low	Low	Low	**Moderate**
Bao (2015)	Low	Unknown	Low	Moderate	Moderate	Low	**Moderate**
Baumann (2009)	Moderate	Unknown	High	Low	High	High	**High**
Benitez-Aguirre (2018)	Low	Low	Low	Low	High	Low	**Moderate**
Broe (2014)	Low	Moderate	Moderate	Low	Low	Low	**Low**
Broe (2014)	Low	Moderate	Low	Low	Low	Low	**Low**
Cankurtaran (2019)	Low	Unknown	Low	Low	High	Low	**Moderate**
Edwards (2005)	Low	Moderate	Low	Low	Low	Low	**Low**
Garcia-Ortiz (2012)	Low	Unknown	Low	High	High	Low	**High**
Garrido (2019)	High	Unknown	Low	Low	High	Low	**High**
Grauslund (2009)	Low	Low	Low	Low	Low	Low	**Low**

Grauslund (2010)	Low	Low	Low	Low	Low	Low	**Low**
Grunwald (2019)	Low	High	Low	Low	Low	Low	**Low**
Gu (2015)	Low	Moderate	Low	Low	Moderate	Low	**Low**
Hwang (2016)	Low	Unknown	Low	Low	Low	Low	**Low**
Keel (2017)	Low	Unknown	Low	Low	Low	Low	**Low**
Klein (2010)	Low	Unknown	Low	Low	Moderate	Moderate	**Moderate**
Liew (2011)	Low	Unknown	Low	Low	High	Moderate	**High**
Lim (2013)	Low	Unknown	Low	Low	Low	Low	**Low**
Mckay (2018)	Low	Unknown	Moderate	Unknown	Moderate	Low	**High**
Mottl (2012)	Low	Low	Low	Low	Low	Low	**Low**
Ooi (2011)	Low	Low	Low	Low	High	Moderate	**Moderate**
Phan (2016)	High	Unknown	High	High	High	High	**High**
Rasmussen (2017)	Moderate	Low	Low	Moderate	Moderate	Low	**Moderate**
Sabanayagam (2008)	Low	Unknown	Low	Low	Low	Low	**Low**
Sabanayagam (2009)	Low	Unknown	Low	Low	Low	Low	**Low**
Sasongko (2012)	Low	Unknown	Low	Low	Moderate	Low	**Low**
Sng (2010)	Low	Unknown	Low	Low	Low	Low	**Low**
Tamadon (2015)	Moderate	Unknown	High	High	High	High	**High**
Vadala (2018)	Moderate	Unknown	Low	Low	High	High	**High**
Wong (2004)	Low	High	Low	Low	Low	Low	**Low**
Zhang (2014)	Low	Unknown	Low	Low	Low	Low	**Low**
Ha M (2019)	Low	Low	Low	Unknown	High	High	**High**
Lee (2014)	Low	Low	Low	Unknown	Moderate	Low	**Low**
